# Editorial: Host Innate Immune Responses to Infection by Avian- and Bat-Borne Viruses

**DOI:** 10.3389/fcimb.2021.651289

**Published:** 2021-02-24

**Authors:** Efstathios S. Giotis, David A. Matthews, Jacqueline Smith

**Affiliations:** ^1^ Section of Virology, School of Medicine, Imperial College London, London, United Kingdom; ^2^ School of Life Sciences, University of Essex, Colchester, United Kingdom; ^3^ School of Cellular and Molecular Medicine, Faculty of Life Sciences, University of Bristol, Bristol, United Kingdom; ^4^ The Roslin Institute and Royal (Dick) School of Veterinary Studies R(D)SVS, University of Edinburgh, Edinburgh, United Kingdom

**Keywords:** avian, zoonotic, virus, innate immunity, bats (Chiroptera)

The COVID-19 pandemic has generated many urgent questions on the origin, trajectory, and host preference of its causative betacoronavirus SARS-CoV-2, as well as renewed focus on other potentially zoonotic viruses. Several species of birds and wild bats can serve as reservoirs and/or mechanical vectors for many infectious viruses including influenza-A, SARS-CoV, MERS, and Ebola. Although substantial progress has been made, there are still major gaps in understanding the emergence, transmission, and adaptation of zoonotic avian- and bat-borne viruses. A major challenge is the dearth of suitable infection and immunity models. Extrapolating data from infection studies in human cell lines or rodents is limiting, as evolutionarily optimized immune factors function differently in non-hosts.

Viral infection triggers anti-viral host defenses and an inflammatory response, broadly coordinated by type I interferon (IFN) and NF-κB, respectively. IFN induces production of hundreds of “interferon-stimulated genes” (ISGs), limiting virus replication until the adaptive immune response can “clean-up.” Rapid initiation of innate immune responses depends on viral recognition by pattern recognition receptors (PRRs) which are expressed in tissues where the virus replicates. Despite the high degree of evolutionary conservation, and assumed similarity in function, there are significant differences between the immune gene repertoires of birds and mammals and bats and other mammals. Ducks utilize many of the mammalian PRRs (i.e. RIG-I, TLR7, and TLR3) and their downstream adaptors to detect and inhibit the replication of influenza viruses (Campbell and Magor). In chickens, key innate immune genes, such as *IRF3* and *RIG-I*, appear to have been lost (Chen et al., 2013). However, chicken cells are capable of initiating an interferon response after virus infection and inducing the expression of hundreds of ISGs (Giotis et al., 2016; Giotis et al., 2017).

Differences in IFN-inducing capacity between virus strains is an important parameter contributing to the host response to viral infection and virulence. Influenza-A virus circulates in multiple hosts causing economic burden in the poultry industry, human epidemics, and occasionally pandemics. Influenza-A virus contains eight negative-strand RNA segments, which are thought to encode 10 viral proteins as well as seven newly identified proteins such as PB1-F2, PA-X whose role is not entirely clear (Ma et al.). The full-length PA-X and PB1-F2 proteins in a recombinant 2009 pH1N1 virus have been reported to enhance viral replication *in vitro*, enhancing viral virulence *in vivo* mainly through simultaneously mediated host innate immune response (Ma et al.).

IBDV (causing Infectious Bursal Disease) is another economically important (but not zoonotic) virus for the poultry industry. Very virulent strains of IBDV (vvIBDV) emerged in the 1980s, causing up to 60% mortality in some commercial flocks for reasons that are poorly understood (Dulwich et al., 2017). Dulwich et al. extended these observations by demonstrating that a vvIBDV strain is able to down-regulate type I IFN and pro-inflammatory cytokine responses compared to a classical field strain both *in vitro* and *in vivo*.

The successful control of virus infection requires the coordination of both the innate and adaptive immune systems. During chronic viral infections the increased level or duration of stimulation of virus-specific CD8 T-cells leads to non-functional state called T-cell exhaustion (Freeman et al., 2006). Recent studies have shown that targeting the immunoreceptor molecule PD1 and its ligand PD-L1 can reverse the exhausted T-cell response (Freeman et al., 2006). The chicken PD-1/PD-L1 might also be used in the treatment of oncogenic avian viruses such as Marek’s disease virus (Reddy et al.).

Current knowledge on bat IFN responses is rudimentary, with most of this obtained in studies of the Australian flying fox (*Pteropus alecto*) (Clayton and Munir). Sequencing of bat genomes suggests that evolution of “metabolically costly” flight imposed adaptations on their innate immune-, DNA damage- and inflammatory-response systems (Zhang et al., 2013). Key antiviral immunity components are conserved in bats, *e.g.* IFNs and their receptors and ISGs However, genes that activate the inflammasome and/or IFN pathways are either missing or have altered function (Clayton and Munir). Studies have inferred a pattern of constitutively expressed IFN-α in unstimulated *Pteropus alecto* cells, which may provide a “switched-on” defense mechanism that blunts virus replication and pathogenesis (Zhou et al., 2016), and can explain why bats are asymptomatic reservoirs of viruses. The remarkable ability of bats to coexist with a wide range of potentially zoonotic viruses is exemplified by the evolutionarily distinct bat influenza-A-like viruses H17N10 and H18N11 (BatIVs). Unlike classical influenza-A viruses, the surface glycoproteins of BatIVs neither bind nor cleave sialic acid receptors, but rather use the trans-species conserved MHC-II proteins (Giotis et al., 2019) to gain cell entry. The scientific evidence so far indicate a limited spillover risk for BatIVs, but data is not conclusive enough to dismiss the possibility of zoonotic transmission (Giotis).

Full-length genome sequences obtained from COVID-19 patients demonstrated that SARS-CoV-2 shares 79.5% sequence identity to SARS-CoV BJ01 strain and a 96% sequence identity to the bat coronavirus RaTG13, suggestive that bats are the original source of the virus (Zhou et al., 2020). The receptor binding protein spike (S) viral gene is highly divergent to other CoVs but has a 93.1% nucleotide identity to RaTG13 (Zhou et al., 2020). Molecular modeling and functional studies revealed that the host surface protein ACE2 mediates cell entry of SARS-CoV-2 and the serine protease TMPRSS2 is essential for S priming (Minakshi et al.). Furthermore, the aftermath of SARS-CoV-2 internalisation is governed by a complex and largely unknown network of host-pathogen interactions. Transcriptomic analysis of human alveolar and bronchial epithelial cells confirmed that the *CSF1* gene, a known target of the microRNA miR-1207-5p, is over-expressed following SARS-CoV-2 infection (Bertolazzi et al.). CSF1 enhances macrophage recruitment and activation and its overexpression may contribute to the acute inflammatory response observed in severe COVID-19.

In conclusion, recent virus outbreaks confirmed the inextricable nature of human and animal health and disease. Interdisciplinary approaches are urgently needed to assess and prevent the spillover of avian- and bat-borne viruses using an organismal biology approach and focusing on viral ecology, diversity, and interactions with the host.

## Author Contributions

EG wrote the first draft and JS and DM edited and commented on the draft. All authors contributed to the article and approved the submitted version.

## Conflict of Interest

The authors declare that the research was conducted in the absence of any commercial or financial relationships that could be construed as a potential conflict of interest.
